# Genes with human-specific features are primarily involved with brain, immune and metabolic evolution

**DOI:** 10.1186/s12859-019-2886-2

**Published:** 2019-11-22

**Authors:** Mainá Bitar, Stefanie Kuiper, Elizabeth A. O’Brien, Guy Barry

**Affiliations:** 1 QIMR Berghofer Medical Research Institute, 300 Herston Road, Herston, QLD 4006 Australia; 2 School of Natural Sciences, Griffith University, Nathan, QLD 4111 Australia; 3 The School of Medicine, The University of Queensland, St Lucia, QLD 4072 Australia

**Keywords:** Human-specific, Brain, Neuron, Glia, Metabolism, Gene expression

## Abstract

**Background:**

Humans have adapted to widespread changes during the past 2 million years in both environmental and lifestyle factors. This is evident in overall body alterations such as average height and brain size. Although we can appreciate the uniqueness of our species in many aspects, molecular variations that drive such changes are far from being fully known and explained. Comparative genomics is able to determine variations in genomic sequence that may provide functional information to better understand species-specific adaptations. A large number of human-specific genomic variations have been reported but no currently available dataset comprises all of these, a problem which contributes to hinder progress in the field.

**Results:**

Here we critically update high confidence human-specific genomic variants that mostly associate with protein-coding regions and find 856 related genes. Events that create such human-specificity are mainly gene duplications, the emergence of novel gene regions and sequence and structural alterations. Functional analysis of these human-specific genes identifies adaptations to brain, immune and metabolic systems to be highly involved. We further show that many of these genes may be functionally associated with neural activity and generating the expanded human cortex in dynamic spatial and temporal contexts.

**Conclusions:**

This comprehensive study contributes to the current knowledge by considerably updating the number of human-specific genes following a critical bibliographic survey. Human-specific genes were functionally assessed for the first time to such extent, thus providing unique information. Our results are consistent with environmental changes, such as immune challenges and alterations in diet, as well as neural sophistication, as significant contributors to recent human evolution.

**Electronic supplementary material:**

The online version of this article (10.1186/s12859-019-2886-2) contains supplementary material, which is available to authorized users.

## Background

Since humans split from the chimpanzee at around 6 million years ago, the different species of the genus Homo (from which modern humans are now the sole representative) have evolved very rapidly, apparently superseding all other events of evolutionary novelty accumulation [1]. Especially prominent differences are observed in aspects such as height, brain size and changes to our gut and skeleton. Environmental alterations such as diet and immune challenges are thought to have played a major role in human-specific adaptations [2, 3]. Although these phenotypic traits, which have a whole-body effect are more readily noticeable, one can easily assume humans have also undergone significant change at the microscopic scale. The question of what makes humans unique at a molecular level is now being more broadly addressed as new and advanced laboratory and bioinformatics tools are enabling comparisons between species from genetic and functional perspectives. Genetic differences between species may have distinct mechanisms of origin, such as alterations in the cytogenetic architecture, local chromosomal rearrangements, gene family duplications, single gene modifications, creations or losses, differences in gene transcription levels and/or patterns and alternative splicing. Functional differences can be observed in general behaviour or tissue and organ development and function, and molecularly in circuits, pathways or cellular variation.

Historically, genomic comparisons in this context date back from the 1970s, when studies comparing humans with non-human primates at the karyotype level were first published, revealing a very close organization of chromosome banding and identical euchromatin [4]. Later, at the chromosome level, translocation and fission events were reported as the first detectable differences between humans and their closest relatives and these were the known genomic landmarks for the origin of Anthropoids [5, 6]. Further, using fluorescent in situ hybridization and comparative genomic hybridization arrays, human-specific segmental duplications and genes displaying human-specific copy number variation were identified [7]. The first human-chimpanzee comparative genome map was published in 2002 and further updated in 2005 [8]. Also in 2005 [9], the first attempt to comprehensively identify human-specific segmental duplications was published from comparisons with the chimpanzee genome, revealing the extent of such alterations, which account for ~ 2.7% of the genomic differences between these species. For comparison, at the nucleotide level, the human and chimpanzee genomes genomes are estimated to differ by > 30 million single substitutions (or ~ 1.2% of the human genome) [8].

Although functional differences between humans and other primates are evident in major morphological features such as the skeleton (e.g. jaws [10] and hands [11]), hair (humans have thinner hair) and muscle tissue [12], and global functions including speech [13] and language [14], changes in the brain have presumably had the most significant impact on the human lineage. The size of the human brain tripled over a period of approximately 2 million years, which overlaps with the estimated period of transition from Australopithecus to Homo [15]. Comparative neuroanatomy has revealed a specific expansion of both the neocortex, with increase in size and neuronal interconnectivity during hominid evolution and the right side of the human brain compared to chimpanzee [16]. While this expansion is believed to be important to the emergence of human language and other high-order cognitive functions, its genetic basis remains largely unknown.

In these last two decades following the first discoveries of genomic differences between humans and other species, numerous studies have identified events that generated human-specific genetic features, such as gene duplications, structural gene alterations and accumulation of significant nucleotide substitutions. Although many authors have worked to identify the genes associated with such human-specific genetic features (hereby referred to as ‘human-specific genes’), no comprehensive and structured list is currently available and the published literature is redundant (in the sense that the same event or gene is many times reported in multiple studies) as well as diverse (in the sense that authors frequently direct their work to different aspects and subsets of genes, thus producing limited results). In summary, current knowledge on the subject is scattered and there is an inherent lack of standard, given the diversity of studies in which one or more human-specific gene is described. Such limitations hinder the study of human-specific genes at a genomic scale, regardless of information being publicly available. Through an extensive bibliographic survey, we gathered, curated and critically assessed the human-specific genes reported in the literature to provide the most comprehensive list to date. We further use this dataset as a platform to explore the general impact of these human-specific genes, assessing their biological impact through functional network and pathway analyses. Finally, we investigate differential gene expression in subpopulations of glial cells and in active versus inactive neurons to examine whether the human-specific genes are involved in specialized neural functions such as cortical development or neuronal activation. Our results highlight the importance of rapid adaptations in immunological, neurological and metabolomic areas that likely contribute to human evolution and identify human-specific genes that are differentially expressed in the brain.

## Results

### The generation of a high confidence structured dataset for human-specific genes

Before describing the obtained results, it is necessary to define our object of study. In this report we use the term human-specific gene when referring to a gene impacted by one or more genetic alterations, which seem to have happened after divergence from non-human primates (usually proposed by genomic comparison with chimpanzee) and result in the emergence of human-specific features. The event causing these genetic alterations may change the gene itself or its regulatory region, as we report in detail.

An extensive bibliographic survey (described within the Materials and Methods section) of the literature published since 2000 resulted on a selective list of 54 scientific articles describing thousands of human-specific features. After triage and manual curation of the data we obtained a set of 982 associated gene descriptors. A descriptor was the most accurate term used by the original author(s) to describe the gene of interest (e.g. name, acronym, database entry number, etc). To standardize notation, for each gene we retrieved information from the human genome version GRCh38. Automatic annotation based on gene descriptor was carried out against the genome and 676 of these genes were directly annotated. Additionally, some gene names contained typos or were slightly modified from their actual name and over 100 other genes had been renamed or restructured since their first annotation. For such genes we carried out manual curation and further annotation when possible. In addition to these individual genes, there are 19 gene families, comprising at least 10 members each, with reported human-specific features that could not be individually attributed to a single gene (Additional file 1 Table S1). Although these gene families were treated separately (to avoid introducing bias given the high number of genes they encompass), when specific genes were described in the literature these were included in the main dataset.

Approximately 130 of the original descriptors could not be associated to any particular gene or gene family, many of these representing genomic fragments as opposed to specific genes and others obsolete or untraceable gene identifiers (IDs). A total of 856 genes (or 871 gene IDs, as some names map to multiple gene IDs, e.g. HAR1A and OR5AL1) with reported human-specific characteristics were curated and annotated and, to the best of our knowledge, comprise the most complete dataset of human-specific genomic features (Additional file 1 Table S1). This number is considerably higher than previously predicted or reported in the literature. For example, the genetics domain of the Matrix of Comparative Anthropogeny (MOCA), which is a repository for available information on human features that differ from great apes, lists only 103 genes known from literature. From these, over 70% are represented in our dataset and most of the remaining were either absent in the current version of the human genome or were filtered out during our manual curation process for lacking strong evidence of human specificity at the gene level.

**Fig. 1:**
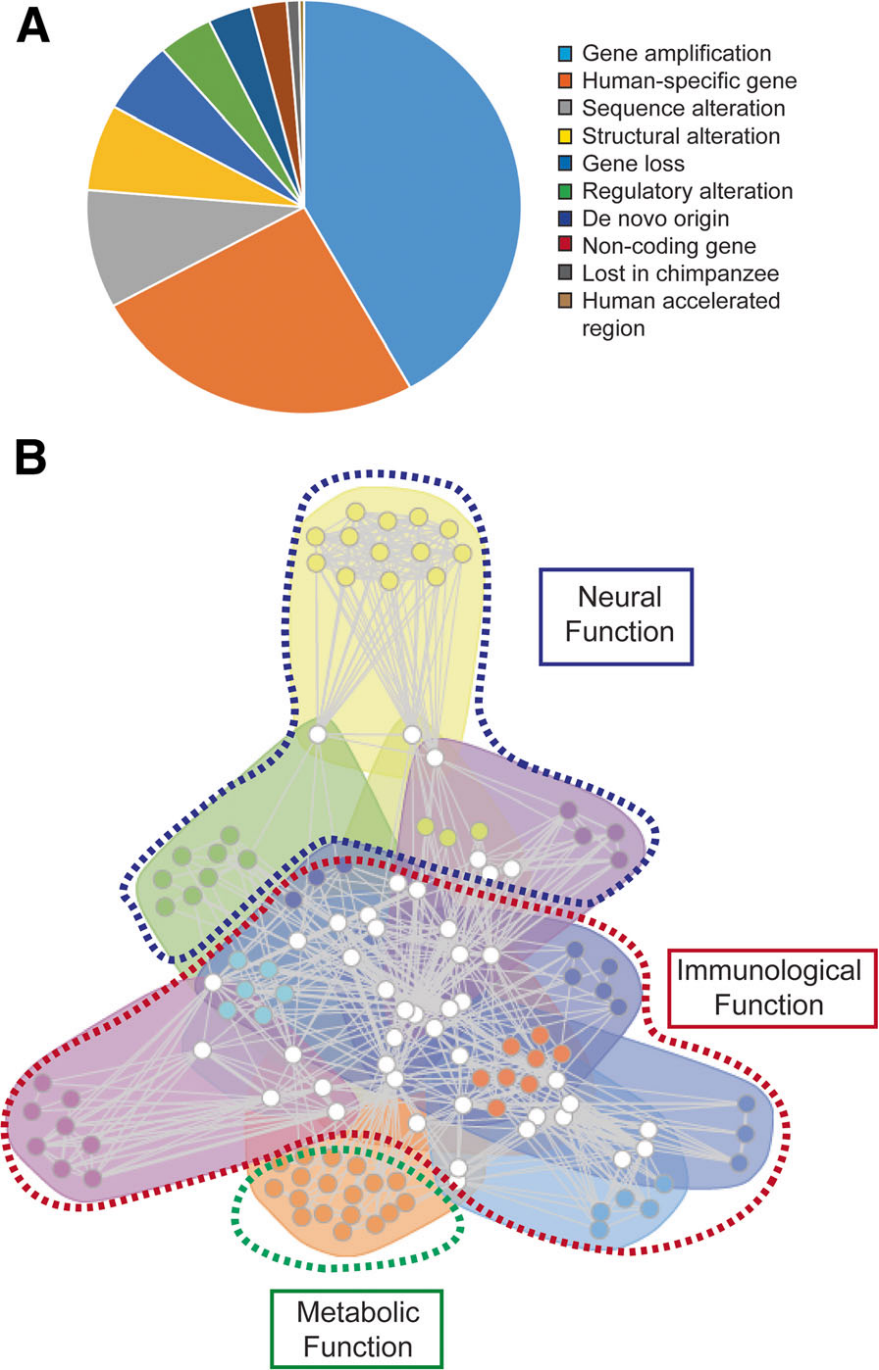
**a** The pie chart illustrates the distribution of 845 genes with human-specific features with regards to the underlying mechanism from which they originated. A more specific classification is shown in the Additional Table 1, as well as additional information for each gene. **b** Functional network of metagroups defined by GeneTerm Linker, represented with FGNet. The 23 metagroups were filtered to allow visualization of metagroups comprising functional terms at organism level and omitting metagroups describing broad cell-level or molecular-level characteristics, which can be assessed from Additional Fig. 1. The color scheme was maintained in all networks to allow comparison

Associated to these genes there are many types of human-specific genetic features and we grouped these in broader classes according to their causative events, also keeping the original description obtained from the correspondent publication from which they were retrieved. All human-specific genes were allocated in one of the 10 following classes (in order of abundance): gene amplification, human-specific gene (undefined feature), gene sequence alteration, gene structure alteration, gene loss, regulatory region alteration, de novo origin, new non-coding gene, lost in chimpanzee and human accelerated region (Fig. 1a). Most genes reported in these articles are protein-coding and thus the resulting database is mainly composed of such genes (588). There are also a large proportion of pseudogenes (186) and non-coding RNAs (55 long ncRNAs and 27 small ncRNAs).

Regarding chromosomal distribution, the 856 genes with human-specific features come from all 22 autosome chromosomes and both sexual chromosomes. No gene was listed from the mitochondrial chromosome. When proportionally compared, the distribution of protein-coding genes with human-specific features and the distribution of all human protein-coding genes per chromosome were relatively similar. A few chromosomes, however, bear a significantly higher number of human-specificity in protein-coding regions. Chromosomes X and 7 seem to be particularly enriched in proteins encoded by genes with human-specific features (Additional file 1 Table S2).

Although this report successfully listed hundreds of genes, it was limited not only by the current availability of studies regarding human-specific genes, but also by poorly defined terminology (the term ‘human-specific’ per se is object of debate, being ambiguously used to describe different levels of specificity). The field itself is specially limited by technical difficulties, such as the lack of a high-quality genome for archaic hominins, complexity of our gene architecture, poorly defined non-coding elements, problems faced when defining genomic correspondence between species, availability of functional data and complications of subsequent validation of predicted variation.

### Functional analyses highlight neuronal, immunological and metabolic features

In possession of the newly generated dataset of genes with human-specific features, we set to investigate the general biological impact that altering their characteristics may have posed to our species. To this end we focus on the functional analysis of each human-specific gene searching further for overall patterns and relationships. Functional enrichment analysis was performed by FGNet [17] using GeneTerm Linker [18] as the underlying algorithm. The resulting network represents the links and associations between metagroups of genes and enriched terms. In total, 295 genes (~ 35%) were successfully functionally annotated by FGNet and assigned to 25 metagroups, two of which were automatically filtered out based on silhouette width. The comprehensive network of metagroups comprising 225 genes is provided as Additional file 1 Figure S1A and the description of each metagroup as Additional file 1 Table S3. Reported *p*-values for all metagroups are lower than 0.0006 (thus orders of magnitude lower than the threshold of 0.05) and each metagroup has at least 10 genes. Since the full network is highly complex, we manually selected 12 metagroups that we trust represent interesting functional classes of systemic level (as opposed to broad molecular or cellular level features). This sub-network clustered into 3 broad functional categories: neural function, immunological function and metabolic function (Fig. 1b and Additional file 1 Figure S1B).

**Fig. 2:**
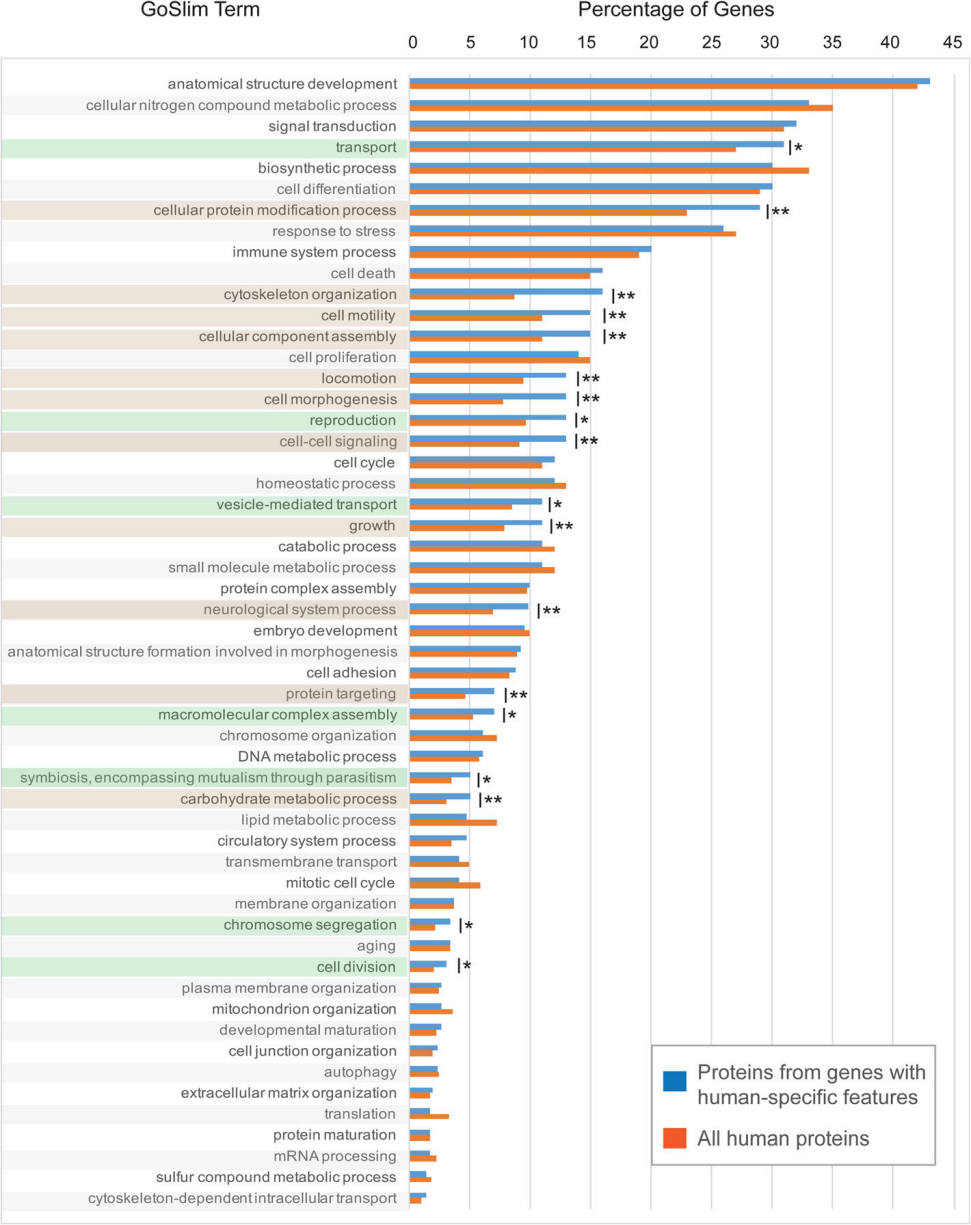
Evidence for enrichment of different gene ontology (GO) terms within the set of genes with human-specific features. Blue bars represent the percentage of genes with human-specific features for which at least one protein product has been associated to a specific GO term. Red bars represent this percentage at the gene level for the entire set of human proteins. Statistical significance of each differentially represented term was assessed using a Fisher exact test. Significant *p*-values are indicated (*p-value ≤0.1; **p-value ≤0.05) and the correspondent term is highlighted in green or brown, respectively

Although FGNet provides a broad overview of the biological impact of human-specific genetic alterations by clustering functional terms in metagroups and establishing relationships between such clusters, it lacks the detail achieved by analyzing each functional class separately. Also, the subset of genes for which GeneTerm Linker could attribute information was only around 35% of the total. Therefore, to examine functional aspects of a higher number of human-specific genes and at a lower scale, we turned to gene ontology (GO) analysis. In total, 596 gene IDs were assigned to at least one human protein sequence, obtained from the Ensembl database (~ 70% of the 871 gene IDs), as a first step for GO annotation. Among the gene IDs for which no protein sequence was retrieved, 187 (~ 70%) are pseudogenes, 84 (~ 30%) are ncRNAs and only 4 are currently annotated as protein-coding (despite no correspondent protein sequences were found). We then assigned functional attributes at the gene level, both for the set of human-specific genes and for the entire set of human proteins, which was used to provide expected abundances. Attributes were assigned to each gene based on the GOSlim catalogue of ontologies for biological processes. We calculated the percentual abundance for each term among human-specific genes and compared with the expected abundance based on observations in all human proteins. Numeric and statistical comparisons indicate the functional terms which are most significantly differentially represented among human-specific genes. Only 3 of the 70 broad GOSlim terms assigned to the entire set of known human proteins were completely absent among the human-specific genes. Among the remaining terms, 11 were significantly over-represented (*p*-values lower than or equal to 0.05) within human-specific genes when compared with the entire set of human proteins (15 other terms had p-values lower than or equal to 0.1; Fig. 2). Enriched terms were involved with neurological system, carbohydrate metabolism, structural growth and functions at the cell level, such as cytoskeleton organization, motility, morphogenesis, locomotion, cell signaling, protein targeting, protein modification and cellular component assembly (Fig. 2). Additionally, interesting terms such as reproduction and symbiosis (encompassing mutualism through parasitism) were highly represented among the human-specific genes, (although their *p*-values were of 0.06 and 0.1, respectively). It is worth mentioning that the term symbiosis in this context was almost entirely related with parasite-host relationships, (50% of the occurrences of this umbrella term related to virus-host interactions) and the term reproduction mostly refers to male reproduction (with 40% occurrences, while the remaining 60% are almost equally shared between female reproduction, general development of the reproductive system and pregnancy-related processes, which encompass fertilization, embryonic and placental development and birth). In summary, based on ontology assignments and subsequent statistical analysis, we highlight that the higher order categories of neural function, carbohydrate metabolism, reproduction and parasite-host relationships are highly correlated with human-specific gene features.

Focusing on pathways as opposed to individual categories or broad clusters of functions, we further analyzed human-specific genes using Ingenuity Pathway Analysis (IPA; [19]). In summary, IPA analysis used 729 out of the 845 genes (~ 85%) and supported the importance of neuronal (e.g. mNOS signaling in neurons, Huntington’s disease signaling), immunological (e.g. phagosome formation, phagocytosis in macrophages) and metabolic (e.g. inositol pyrophosphates biosynthesis, adipogenesis pathway, glutamate biosynthesis and degradation) functions (Additional file 1 Figure S2). Taken together multiple functional analyses tools have converged to generally implicate neuronal, immunological and metabolic systems with human evolution and species-specific characteristics.

### Highly expressed human-specific genes are cell-type enriched across different radial glial cell populations

**Fig. 3:**
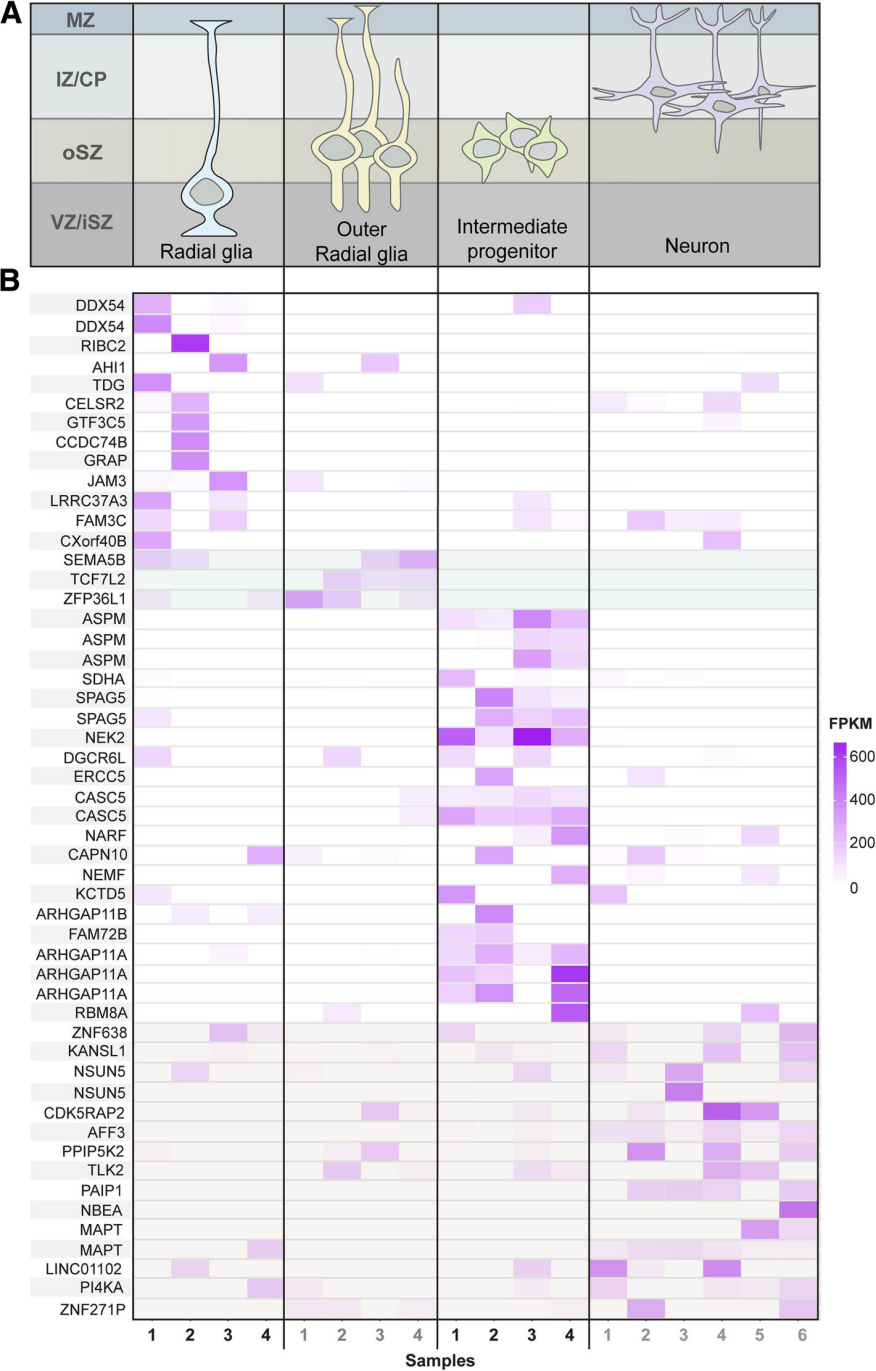
**a** The upper panel depicts differentiation stages of human radial glia cells. Rows represent the correspondent brain location and columns represent the different cell types along a virtual timeline. Cell location starts from the inner layer of the ventricular zone (the inner subventricular zone VZ/iSZ) and goes through the outer subventricular zone (oSZ) to the region between the intermediate zone and the cortical plate (IZ/CP). **b** The heatmap is a graphical representation of normalized read counts (FPKM values) across all samples for each transcript of highly expressed genes with human-specific features. Color intensity varies according to FPKM value as shown in the scale. Highly expressed genes were defined as the top 2000 in terms of FPKM and those that were highly expressed in at least one of the four cell stages were selected. The first 12 genes are highly expressed in radial glial cells, the next 3 genes (light grey shade) in outer radial glia, the further 15 in intermediate progenitor cells and the last 13 (light grey shade) in differentiated neurons. The columns of the heatmap represent different samples of each cell type (the 4 radial glia samples, 4 outer radial glia samples, 4 intermediate progenitor samples and 6 neuron samples)

Since the human brain has such remarkable properties, with many cognitive traits being postulated to be unique to our species [20], we turned to investigate the unique expression profile of human-specific genes within glial cell subpopulations (which ensure homeostasis and provide support and protection to neuronal cells in the brain). The cell populations we selected as object of study are distinctively located at the subventricular zone, a well known center for neuronal cell production in primates. The expression of human-specific genes in such location could be related with the unique enlargement and folding of the human brain, driven by neocortical expansion (see [21] for further information). Using publicly available samples retrieved from the Sequence Read Archive (SRA) we have assessed transcript abundance for the set of human embryonic radial glial cells, outer radial glial cells, intermediate progenitor cells and neuron cells (study SRP094417). We used FPKM (Fragments Per Kilobase of transcript per Million mapped reads) values calculated with RSEM as expression measures. A consistent number of transcripts was shown to be expressed (at any level) across all 4 sets of samples and these represent approximately 10% of the ~ 200,000 transcripts in the reference transcriptome. We defined highly expressed transcripts as the top ~ 10% of the expressed transcripts, i.e. the 2000 transcripts with highest average FPKM values for each set of samples. Highly expressed transcripts were then mapped to their gene of origin (on average ~ 1580 genes were characterized as highly expressed) and compared with the set of 856 genes with human-specific features. We retrieved 23 highly expressed human-specific genes from the radial glial cell samples, 17 from the outer radial glial cell samples, 26 from the progenitor cells samples and 24 from the neuron cell samples. The list of transcripts related to these human-specific genes as well as their estimated expression in each cell population is available as Additional file 1 Table S4. From the non-redundant total of 61 genes overall, 43 (> 70%) were highly expressed in a cell-specific manner (Fig. 3). The heatmap represents expression levels for the set of transcripts associated with these 43 genes (which generate 52 transcripts) across all 4 cell populations (Fig. 3b). We thus have uncovered sets of human-specific genes for which all transcripts are highly expressed in specific cells and have very low expression across all other 3 cell types (i.e. are virtually cell-specific). Many of these genes have been previously implicated with human phenotypes, including developmental delay (e.g. ASPM [22], AFF3 [23] and MAPT [24]) and intellectual disability (e.g. NEMF [25], PI4KA [26] and KANSL1 [27]).

### Multiple human-specific genes are differentially expressed upon activation in neurons derived from induced pluripotent stem cells (iPSC)

**Fig. 4:**
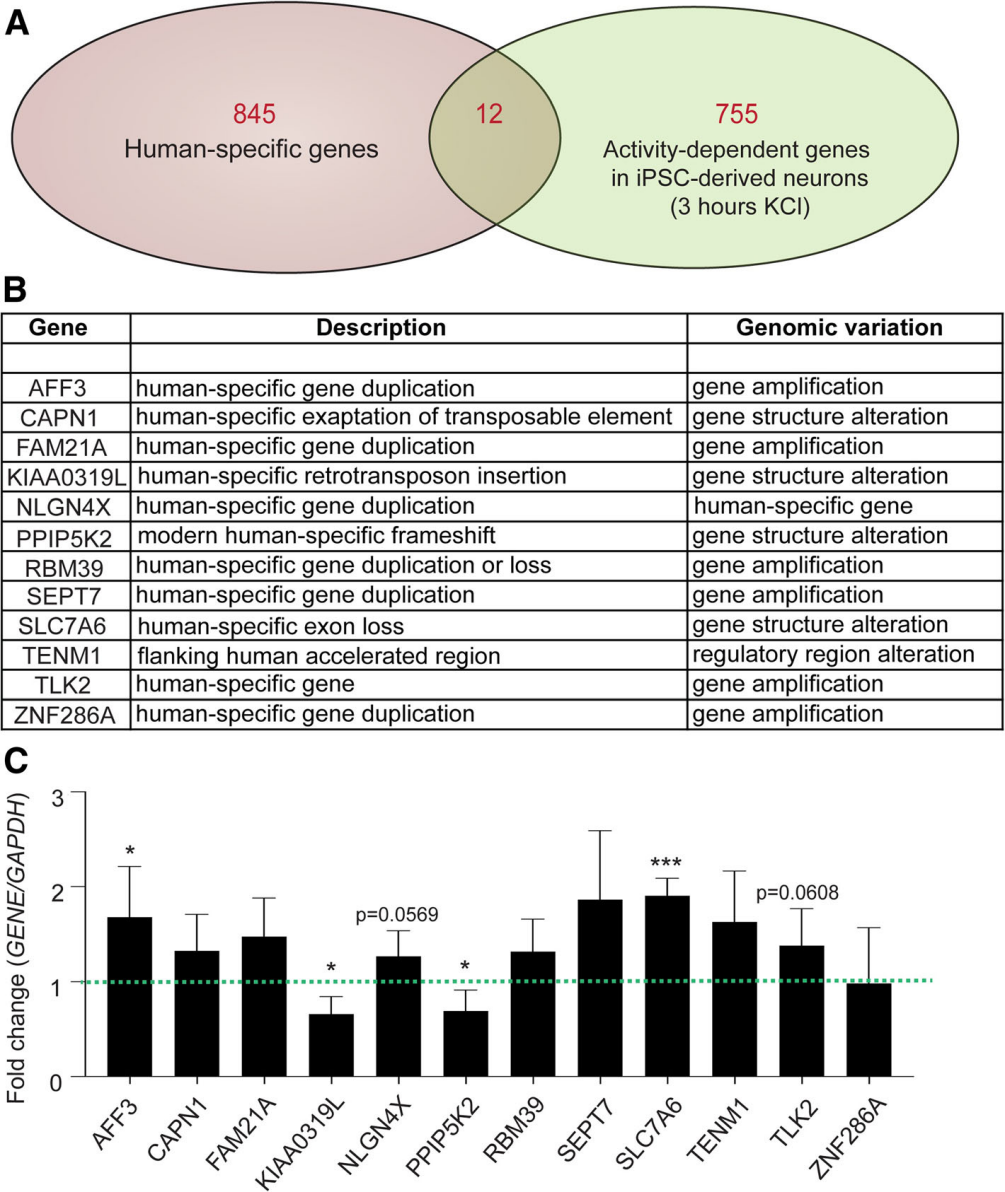
**a** The diagram represents the intersection between the set of genes with human-specific features and the set of genes that undergo differential expression upon activation of iPSC-derived neurons with KCl for 3 h. **b** The 12 human-specific differentially expressed genes and their mechanism of origin. **c** Gene expression was investigated by RT-qPCR using RNA from the same samples of active and inactive iPSC-derived neurons. Fold-change values were calculated relative to *GAPDH* expression. Statistical significance was performed using unpaired t-test (*p-value < 0.05; ***p value < 0.001)

As another example of roles human-specific genes may perform in the brain, we carried out RNA-Seq analyses of neurons differentiated from human iPSC before and after cell activation (50 mM KCl for 3 h) to investigate differential expression of human-specific genes upon neuronal activation. As a result, 798 transcripts were shown to be differentially expressed, 407 being under-expressed upon activation and 391 over-expressed. These transcripts correspond to 755 genes, 12 of which have human-specific features (Fig. 4a, b). These 12 genes have multiple roles and some are implicated in synaptic function (e.g. SEPT7 [28] and CAPN1 [29]) and neurological diseases (e.g. AFF3 [30], NLGN4X [31], CAPN1 [32] and KIAA0319L [31]). We performed RT-qPCR to validate the expression profile of these genes and found 4 of these to be significantly altered after 3 h KCl activation AFF3, KIAA0319L, PPIP5K2 and SLC7A6 (Fig. 4c).

## Discussion

We set out to survey the scientific literature for genes previously reported as human-specific, knowing a better understanding of how these genes have mechanistically impacted our evolution would be broadly beneficial for the study of human physiology and disease. The resulting dataset of genes associated with human-specific variants is, to the best of our knowledge, the most detailed, structured and comprehensive to date. Here we highlight higher order functional areas which house a large number of human-specific genes and are likely to by impacted by these genes and their products. Functional assessment of more than 850 human-specific genes emphasized the significance of brain, immune and metabolic adaptations.

In hindsight these findings may not be completely unexpected as infections, dietary alterations (coincident with the discovery of tools and the domestication of fire for cooking) and extraordinary brain expansion have been well documented.

Although humans possess a great degree of plasticity for adaptation, it is likely that the real origin of the human adaptations that truly ignited human uniqueness occurred during the time of Australopithecus and early Homo species [33, 34]. At this time there was widespread movement, the emergence of tools, an enlargement of the brain and a decrease in masticatory apparatus relative to an increasing body size. The human brain has evolved rapidly in the past 2 million years (coincidental with the emergence of Homo species) and continues to do so through highly unstable, or rather adaptable, regions in our genome, tissue-specific and function-specific gene expression and reorganized circuitry [35]. Nevertheless, it was very likely a conjunction of factors that enabled human evolution to occur at such a rapid rate. For example, newly formed regions of the human brain such as the prefrontal cortex seem to have far higher energy requirements than more conserved regions [36]. It may be that it was only possible to meet such requirements through modifications to food preparation methods that ultimately resulted in higher energy intake [37]. This example could illustrate a crosstalk between different aspects of human evolution which may have resulted in emergent properties of our species. Significant changes are also observed in local adaptations in recent human populations to environmental and behavioral factors such as diet, infections, altitude and temperature [38]. Emerging pathogens that specifically infect humans have to some degree been impacted by our own innovations, such as agriculture, and continue to shape our immune evolution through host-pathogen interactions [39].

## Conclusions

Despite limitations, our comprehensive study contributes to the current knowledge by considerably updating the number of human-specific genes and further emphasizing the importance of brain, immune and metabolic adaptation in defining our species. It also highlights the potential significance of considering metabolism in conjunction with brain function to fully understand human-specific function and disease.

## Materials and methods

### Database of genes with human-specific features

We have extensively scanned and curated the current literature and searched for articles describing human-specific genetic features and its associated genes. PubMed (www.ncbi.nlm.nih.gov/pubmed) was used as the search platform with the criteria “Search human specific gene Filters: Publication date from 2000/01/01 to 2017/12/31” (further expanded to 2019/12/31), which resulted in over 218,000 publications. From these articles, we selected for terms such as “human-specific”, “duplication”, “de novo”, “evolution” among other terms of interest. Studies were also assessed regarding their relevance/direct relation to the topic, design of the study, type of publication and whether or not the publication was peer reviewed. An initial subset of 36 highly relevant and non-redundant studies were selected and further expanded (mainly through citation relationships) to 54 references from which data were retrieved. These articles report human-specific genetic features, i.e. gene-related molecular characteristics that have been reported to differ between humans and other species and are likely to impact the associated gene (such as changes to the sequence of a gene promoter, exon losses, gene duplications, etc). The genetic features are related to specific genes, which are the object of study of the present work. Gene names were listed and duplicated entries were collapsed. Ambiguities were assessed in as much detail as possible to clarify the specific gene authors referred to. The initial list was mapped back to the GRCh38 version of the human genome and remaining non-annotated entries mainly represented genes that have been renamed or excluded since their first annotation. The final set of genes was categorized according to the reported human-specific feature and grouped by biotypes as proposed in the Ensembl glossary (publicly available at ensembl.org/Help/Glossary).

### Chromosomal distribution of human-specific protein-coding genes

There are 596 gene IDs associated with protein-coding genes. These were listed regarding their chromosome of origin and the proportion of entries per chromosome was calculated. The same was performed with the entire set of protein-coding genes annotated in the human genome, for comparison. In parallel, we used the GeneOverlap library (version 1.12.0) of the R package to infer significance of overlapping genes. The internal algorithm for Fisher’s exact test used by this package determined the respective *p*-values (which were not corrected for multiple hypothesis).

### Functional analysis of genes with human-specific features

Genes were also subject to functional analyses for the generation of a protein-protein interaction network and functional clusterization using the Bioconductor package FGNet version 3.10.0 [17] and GeneTerm Linker [18] for functional enrichment analysis. Metagroups with silhouette width of less than 0 were excluded and a minimum support of 3 genes was required for cluster validation.

Human protein sequences were obtained from Ensembl GRCh38 [40] and genes with human-specific features had their respective protein sequence(s) retrieved. The retrieved sequences were submitted to AgBase GoAnna version 2.0.0 [41] for GO assignment based on sequence homology. Blastp was used as the underlying algorithm and search parameters were an E-value cutoff of 10e-50, BLOSUM62 as the substitution matrix, a minimum of 80% sequence identity plus 75% coverage and default word size and gap penalty values. GoAnna results were submitted to AgBase GOSlim [41] to obtain high-level summaries of functions for the given dataset and further analyses were restricted to categories of biological processes, which involve pathways and activities of multiple genes. The same protocol was used to assign GOSlim terms to the entire set of human proteins obtained from Ensembl. Results report the percentual of each term both in the set of human-specific proteins and all human proteins, which was used as background. Against this background of expected abundance, significance for differential representation of functional terms within the human-specific subset of proteins was calculated using Fisher’s exact test (implemented in the GeneOverlap library of the R package version 1.12.0) to determine the respective *p*-values (which were not corrected for multiple hypothesis).

### SRA samples of radial glial cells

We retrieved fastq files from the SRA-deposited study SRP094417, which contains 18 runs from samples of prenatal human brain, representing data with replicates from radial glial cells, outer radial glial, intermediate progenitor and mature neuronal cells. Reads are paired-end and were generated from cDNA with the Illumina HiSeq2000 platform in 2016.

### RNA-Seq of iPSC

The generation and activation of human iPSC-derived neurons and RNA isolation, preparation and sequencing were described in a previous report by our group [42].

### RNA-Seq analysis

Both the set of iPSC and SRA-retrieved RNA-Seq samples were treated with the same bioinformatics pipeline, which is composed of 5 main steps: (1) Pre-trimming quality control with FastQC version 0.11.5 (bioinformatics.babraham.ac.uk/projects/fastqc); (2) Read trimming with Trimmomatic version 0.36 [43]; (3) Post-trimming quality control with FastQC; (4) Alignment or pseudoalignment to reference transcriptome and read counting for transcript abundance estimation with Kallisto version 0.43.0 or STAR-RSEM versions 2.5.2a and 1.2.30 [44–46]; (5) Measurement of differential expression of transcripts with EdgeR version 3.18.1 [47]. Each step is generally described below.

FastQC was used for quality control of raw reads and a comparative round of quality control after running Trimmomatic, to ensure overall quality was either maintained or increased after read trimming. The set of default parameters was used for this step. Trimmomatic was employed for cleaning reads from sequencing artifacts. The set of Illumina adapters for the TruSeq paired-end library preparation kit was used as database for adapter trimming. Reads were scanned with a 4-base wide sliding window and trimmed when the average quality per base was lower than 20. Reads shorter than 40 bases after trimming were further excluded. Kallisto and STAR-RSEM were used as different alternatives to generate read counts. Kallisto performs pseudoalignments and read counts within the same command line, while STAR performs alignments to the reference transcriptome and the result is used by RSEM to generate read counts. Kallisto indexing tool was used to generate an index for the FASTA formatted file of the human transcriptome with k-mer size of 31. Reads were counted for transcript quantification using default parameters and a number of bootstrap samples of 100. As an alternative to estimate transcript abundance, STAR was used to perform alignments between the paired-end reads and the reference human transcriptome. An index was built with default parameters and the alignment was performed discarding multimappers and defining parameters for splicing treatment. Resulting bam alignment files were further converted to sam files using Samtools (samtools.sourceforge.net) and sorted with Novosort (novocraft.com/products/novosort), as an intermediate step. RSEM was used to prepare a reference file from the human transcriptome and count reads to provide transcript abundance in the paired-end mode. EdgeR was used to perform statistical analysis and define differentially expressed genes. Kallisto and STAR-RSEM results were compared to evaluate data robustness. In summary, when results were qualitatively similar, parameters were considered well adjusted. After assessing different thresholds, a minimum of 5 reads per transcript before normalization was needed to validate expression. Read counts generated by STAR-RSEM were used for differential expression assessment. Samples were normalized based on sample sizes and data variability was estimated according to a negative binomial dispersion parameter. Differential expression was reported with limits being a*p*-value of less than 0.001 and false discovery rate of less than 0.01.

### Quantitative RT-PCR for differentially expressed human-specific genes in iPSC data

Quantitative RT-PCR was used to validate expression patterns for the subset of genes with human-specific features shown to be differentially expressed in iPSC. cDNA synthesis was performed using the SuperScript III First-Strand Synthesis System (ThermoFisher Scientific, USA). Briefly, 500 ng of total RNA was used and random hexamer primed protocol was followed. Each cDNA sample was amplified in triplicate using SYBR Green PCR Master Mix (ThermoFisher Scientific, USA). Primer pairs used for this analysis are described in Additional file 1 Table S5.

## Additional file

Additional file 1: **Table S1.** The screenshot above represents the first lines of the table. The full version is given as an independent Supplementary Table in xls format. This file contains all 845 genes with humanspecific features retrieved in this study (Sheet 1 - "HumanSpecific genes") and describes for each gene its: (A) Gene name (updated to the current Ensembl description, when necessary); (B) Ensembl ID; (C) Chromosome number; (D) Gene type (the specific type, as described by Ensembl); (E) General gene type (a general classification which may group multiple gene types - e.g. pseudogenes includes processed, unprocessed and transcribed pseudogenes); (F) Mechanism of origin (specifically as described by the author of the correspondent reference); (G) General mechanism of origin (a manually assigned general classification which may group multiple subclasses from column (F) - data from this column was used to generate the pie chart presented in Figure 1a in the main manuscript) and (H) At least one reference in which the gene is reported (the full list of references, numbered accordingly, is given as Sheet 2 - "References"). The file also contains information on 19 large gene families (Sheet 3 - "HumanSpecific GeneFamilies"), described as undergoing significant expansion or accelerated evolution across all (or many of) its members. These were not included in the main table, mainly to prevent their high gene numbers to introduce a functional bias in the dataset. **Table S2.** This table presents the percentage of protein-coding genes in each chromosome (A), both for the set of genes with human-specific features (B) and the entire set of human proteins retrieved from the Ensembl database (C). A p-value is given (D), generated with a Fisher's exact test to represent the significance of the difference between (B) and (C) per chromosome. Chromosomes X and 7 are clearly enriched in genes with human-specific features and another four (in green) have significantly more of such genes than expected. **Table S3.** This table describes the metagroups generated by GeneTerm Linker using FGNet. The metagroup number corresponds to numbers in Supplementary Figures 1A and 1B (and information can be transferred to Figure 1B in the manuscript). For each metagroup this file presents its silhouette size (a clustering coefficient), significance (p-value), number of constituent genes and constituent functional terms (or, for metagroups 1 and 6, which do not meet inclusion cutoffs, their exclusion criteria). The last column on the right describes functional terms in each metagroup and their annotation space, which can be a gene ontology assignment (GO for biological process, molecular function or cellular component), a KEGG pathway or a function inferred from the description of InterPro motifs or domains (IPR).**Table S4.** This table presents gene expression levels (in FPKM) for transcripts related to 61 humanspecific genes which were characterized as highly expressed in at least one subpopulation of glial cell (sequencing data retrieved from SRA). Highly expressed transcripts were defined as the top ~10% of the expressed transcripts (i.e. the 2,000 transcripts) with highest average FPKM values for each set of samples under investigation. On average ~1580 genes were characterized as highly expressed in each cell type and compared with the set of 856 genes with human-specific features. We retrieved 23 highly expressed human-specific genes from the radial glial cell samples, 17 from the outer radial glial cell samples, 26 from the progenitor cells samples and 24 from the neuron cell samples, resulting in a set of 61 nonredundant genes and 91 transcripts. **Table S5.** Primers designed for each of the 12 genes described in the Figure 4 of the main manuscript. Gene names and accession numbers are also provided.**Figure S1**. These functional networks describe the set of genes with human-specific features. The network outputs were generated with FGNet to represent the metagroups defined with GeneTerm Linker. Individual files are provided to allow assessment of gene names and network topology. A) From a total of 25 metagroups (clusters of associated genes with coherent biological significance), 2 were filtered out for not meeting the parameters for measuring relevance, in terms of significance and coherence. The remaining 23 metagroups represent 225 genes and many different functions, including neuronal, metabolic and immunological. Metagroups are color-coded and their full description is given in the Supplementary Table 3. Gene names and network topology can be better visualized when magnified. White circles denote genes shared by multiple metagroups. **Figure S2.** The Ingenuity Pathway Analysis (IPA) is an additional tool for functional analysis of highthroughput sequencing data. In this figure we present results generated using IPA for the set of genes with human-specific features. This result includes >85% of the 845 genes in the dataset and describes these in terms of the pathways in which they function. A) The plot presents category scores. The "threshold" line (vertical line in light orange, set here to 1.25) indicates the minimum significance level in terms of inverse logarithmic *p* values [-log(p-value)] derived from performing a Fisher’s exact test. The proportion of genes in the dataset that map to each pathway in the IPA knowledgebase is represented as the "ratio" (line in darker orange). The z-score is color-coded and refers to the difference between observed and predicted up/down regulation states of pathways. B) The table shows higher-order functional classes with their respective significance (*p* values) and the number of genes (molecules) by which they are composed. (PDF 2947 kb)

Additional file 2 (XLSX 140 KB)

## Data Availability

Authors state that all data used to generate the set of human-specific genomic regions can be found within the manuscript text and/or at the Additional Material (mainly in Additional file 1 Table S1).

## Abbreviations

FPKM: Fragments Per Kilobase of transcript per Million mapped reads
GO: gene ontology
IDs: identifiers
IPA: Ingenuity Pathway Analysis
iPSC: induced pluripotent stem cells
MOCA: Matrix of Comparative Anthropogeny
ncRNAs: non-coding RNAs
SRA: Sequence Read Archive
