# Supplementary material for: Genes with human-specific features are primarily involved with brain, immune and metabolic evolution
Source: BMC Bioinformatics. 2019 Nov 22;20(Suppl 9):406. doi: 10.1186/s12859-019-2886-2 (PMC6873653; doi:10.1186/s12859-019-2886-2)
Supplement: Supplementary file 1 — Table S1. The screenshot above represents the first lines of the table. The full version is given as an independent Supplementary Table in xls format. This file contains all 845 genes with humanspecific features retrieved in this study (Sheet 1 - "HumanSpecific genes") and describes for each gene its: (A) Gene name (updated to the current Ensembl description, when necessary); (B) Ensembl ID; (C) Chromosome number; (D) Gene type (the specific type, as described by Ensembl); (E) General gene type (a general classification which may group multiple gene types - e.g. pseudogenes includes processed, unprocessed and transcribed pseudogenes); (F) Mechanism of origin (specifically as described by the author of the correspondent reference); (G) General mechanism of origin (a manually assigned general classification which may group multiple subclasses from column (F) - data from this column was used to generate the pie chart presented in Figure 1a in the main manuscript) and (H) At least one reference in which the gene is reported (the full list of references, numbered accordingly, is given as Sheet 2 - "References"). The file also contains information on 19 large gene families (Sheet 3 - "HumanSpecific GeneFamilies"), described as undergoing significant expansion or accelerated evolution across all (or many of) its members. These were not included in the main table, mainly to prevent their high gene numbers to introduce a functional bias in the dataset. Table S2. This table presents the percentage of protein-coding genes in each chromosome (A), both for the set of genes with human-specific features (B) and the entire set of human proteins retrieved from the Ensembl database (C). A p-value is given (D), generated with a Fisher's exact test to represent the significance of the difference between (B) and (C) per chromosome. Chromosomes X and 7 are clearly enriched in genes with human-specific features and another four (in green) have significantly more of such genes than expecte [file 12859_2019_2886_MOESM1_ESM.pdf]

__SUPPLEMENTARY MATERIAL__

Human-specific genomic variants associated with protein-coding genes suggest significant brain, immune and metabolic evolution.

Mainá Bitar[1], Stefanie Kuiper[1], Elizabeth A. O'Brien[1], Guy Barry[1,2]*

[1]QIMR Berghofer Medical Research Institute, Herston, QLD 4006, Australia.
[2] The School of Medicine, The University of Queensland, St Lucia, QLD 4072, Australia.

***Corresponding author**
Dr Guy Barry
QIMR Berghofer Medical Research Institute
300 Herston Road, Herston
QLD 4006, Australia
Telephone: +61 7 3362 0138
Fax: +61 7 3362 0111
Email: Guy.Barry@qimrberghofer.edu.au

Supplementary Figure 1 – Functional Networks of Genes with Human-Specific Features

A

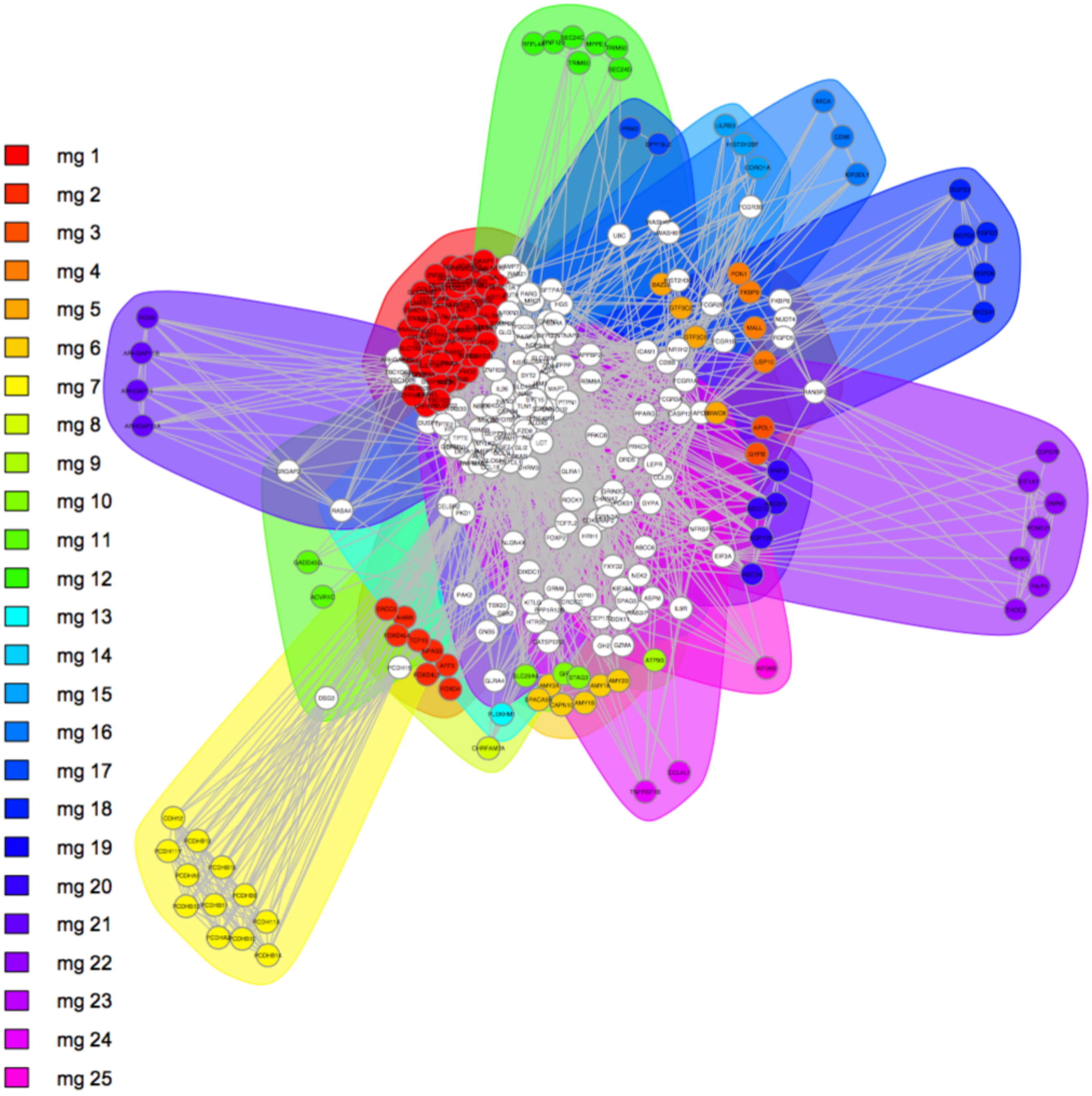

B

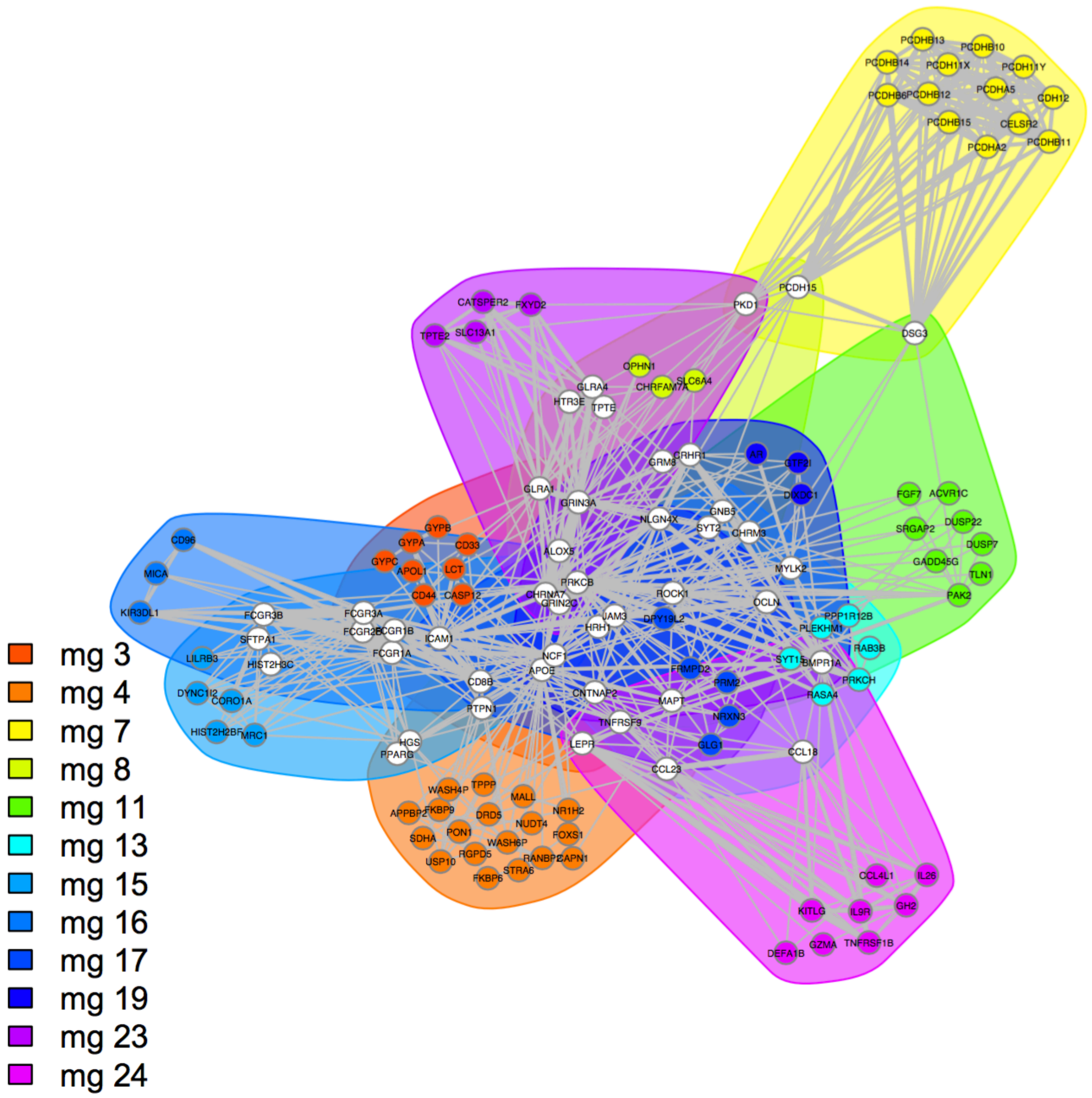

Supplementary Figure 1:
These functional networks describe the set of genes with human-specific features. The network outputs were generated with FGNet to represent the metagroups defined with GeneTerm Linker. Individual files are provided to allow assessment of gene names and network topology.
A) From a total of 25 metagroups (clusters of associated genes with coherent biological significance), 2 were filtered out for not meeting the parameters for measuring relevance, in terms of significance and coherence. The remaining 23 metagroups represent 225 genes and many different functions, including neuronal, metabolic and immunological. Metagroups are color-coded and their full description is given in the Supplementary Table 3. Gene names and network topology can be better visualized when magnified. White circles denote genes shared by multiple metagroups.
B) The full network described in Figure 1A was filtered to allow for a figure within the main manuscript. This is the higher-resolution version of the manuscript Figure 1B. The color-code and numbering of metagroups was maintained to allow comparison between figures. Metagroups are color-coded and their full description is given in the Supplementary Table 3. Gene names and network topology can be better visualized when magnified.

Supplementary Figure 2 – Pathway Analysis of Genes with Human-Specific Features

A

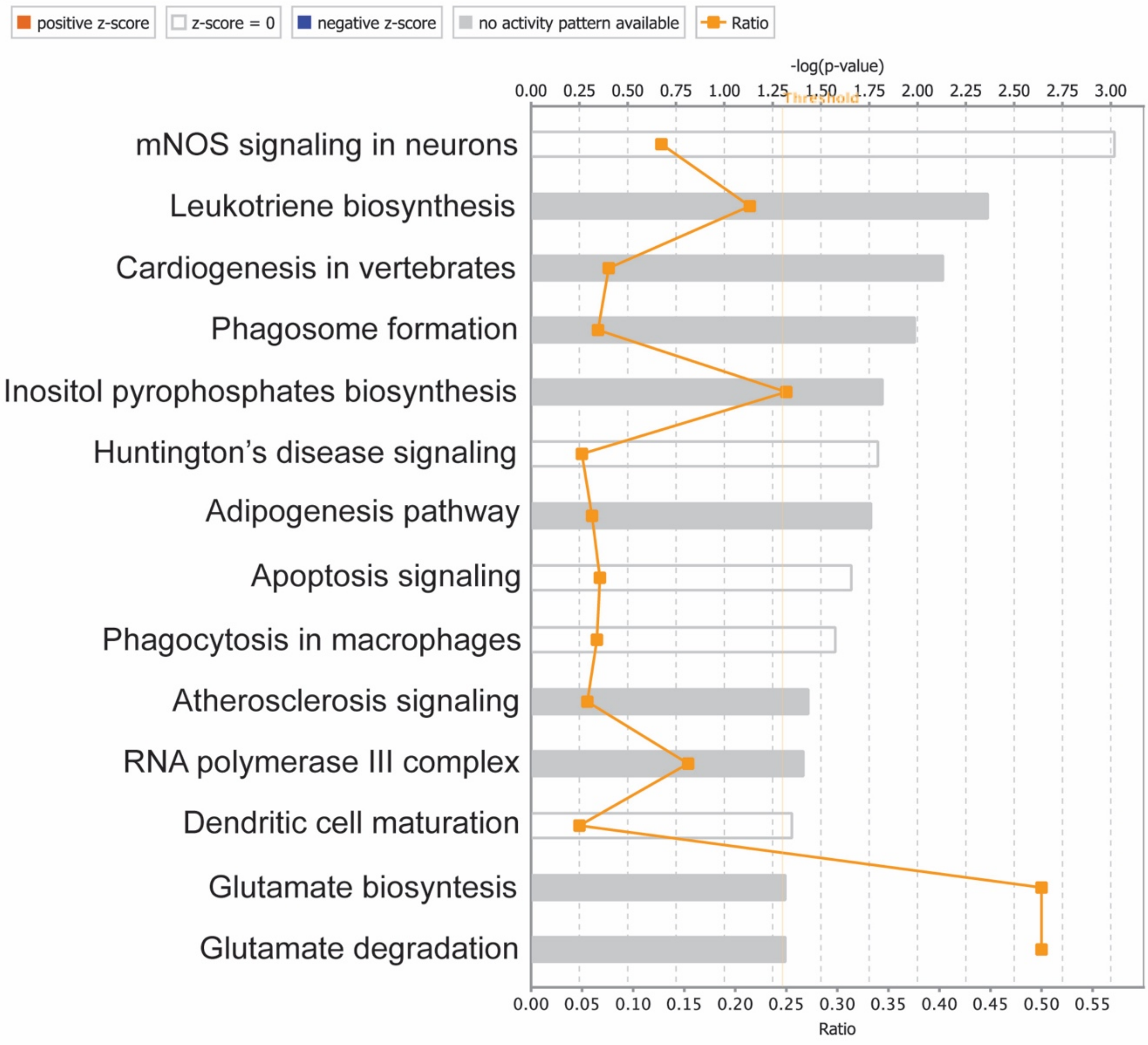

B

Physiological System Development and Function

Name	p-value range	Molecules
Nervous System Development and Function	7.36E-03 - 9.30E-07	71
Digestive System Development and Function	7.84E-03 - 4.340E-06	33
Hepatic System Development and Function	7.74E-03 - 4.34E-06	20
Organ Development	7.74E-03 - 4.34E-06	31
Tissue Morphology	8.40E-03 - 2.30E-05	58

Supplementary Figure 2:
The Ingenuity Pathway Analysis (IPA) is an additional tool for functional analysis of high-throughput sequencing data. In this figure we present results generated using IPA for the set of genes with human-specific features. This result includes >85% of the 845 genes in the dataset and describes these in terms of the pathways in which they function.

A) The plot presents category scores. The "threshold" line (vertical line in light orange, set

here to 1.25) indicates the minimum significance level in terms of inverse logarithmic p-values [-log(p-value)] derived from performing a Fisher's exact test. The proportion of genes in the dataset that map to each pathway in the IPA knowledgebase is represented as the "ratio" (line in darker orange). The z-score is color-coded and refers to the difference between observed and predicted up/down regulation states of pathways.

B) The table shows higher-order functional classes with their respective significance (p-values) and the number of genes (molecules) by which they are composed.

Supplementary Table 1 – Genes with Human-Specific Features

A	B	C	D	E	F	G	H
**Gene Name**	**Ensembl ID**	**Chromosome**	**Gene Type**	**General Gene Type**	**Mechanism of Origin**	**General Mechanism of Origin**	**Reference(s)**
AATBC	ENSG000002	21	antisense	long non-coding	hominoid-specific de novo originated protein-coding gene	de novo origin	1
ABCB10	ENSG000001	1	protein_coding	protein-coding	human-specific gene amplification	gene amplification	2,3
ABCC12	ENSG000001	16	protein_coding	protein-coding	modern human-specific coding change	gene sequence alteration	4
ABCC6	ENSG000000	16	protein_coding	protein-coding	human-specific gene duplication or expansion	gene amplification	5
ABCD1P2	ENSG000002	10	unprocessed_pseudogene	pseudogene	human-specific gene	human-specific gene (undefined feature)	6
ABCD1P5	ENSG000002	2	unprocessed_pseudogene	pseudogene	human-specific gene	human-specific gene (undefined feature)	6
ABCD4	ENSG000001	14	protein_coding	protein-coding	modern human-specific stop loss generating NMD transcript	gene structure alteration	4
ABHD17A	ENSG000001	19	protein_coding	protein-coding	human-specific gene duplication	gene amplification	7
ABHD17AP1	ENSG000001	1	unprocessed_pseudogene	pseudogene	human-specific gene	human-specific gene (undefined feature)	6
AC005042.4	ENSG000002	2	antisense	long non-coding	human-specific de novo originated protein-coding gene	de novo origin	8
AC005488.1	ENSG000002	7	unprocessed_pseudogene	pseudogene	human-specific gene	human-specific gene (undefined feature)	6
AC008132.1	ENSG000001	22	transcribed_unprocessed_pse	pseudogene	human-specific gene	human-specific gene (undefined feature)	6
AC008132.1	ENSG000001	22	lincRNA	long non-coding	human-specific gene	human-specific gene (undefined feature)	6
AC026271.5	ENSG000001	17	processed_pseudogene	pseudogene	human-specific gene	human-specific gene (undefined feature)	6
AC027612.6	ENSG000001	2	transcribed_unprocessed_pse	pseudogene	human-specific gene	human-specific gene (undefined feature)	6
AC062028.1	ENSG000001	2	lincRNA	long non-coding	hominoid-specific de novo originated protein-coding gene	de novo origin	1
AC083899.3	ENSG000002	2	unprocessed_pseudogene	pseudogene	human-specific gene	human-specific gene (undefined feature)	6
AC096670.3	ENSG000002	2	antisense	long non-coding	human-specific de novo originated protein-coding gene	de novo origin	8
AC111200.7	ENSG000002	2	processed_pseudogene	pseudogene	human-specific gene duplication	gene amplification	7
AC129778.2	ENSG000001	9	lincRNA	long non-coding	human-specific gene	human-specific gene (undefined feature)	6
AC138969.4	ENSG000001	16	protein_coding	protein-coding	human-specific gene	human-specific gene (undefined feature)	6
ACTR3B	ENSG000001	7	protein_coding	protein-coding	human-specific gene duplication	gene amplification	9
ACVR1C	ENSG000001	2	protein_coding	protein-coding	human-specific inactivation by retrotransposon insertion	gene loss	10
ADARB1	ENSG000001	21	protein_coding	protein-coding	human-specific exon gain	gene structure alteration	11,12
ADORA2A-A	ENSG000001	22	antisense	long non-coding	hominoid-specific de novo originated protein-coding gene	de novo origin	13,1
AFF3	ENSG000001	2	protein_coding	protein-coding	human-specific gene duplication	gene amplification	9
AGR3	ENSG000001	7	protein_coding	protein-coding	human-specific gene amplification	gene amplification	47
AGAP1	ENSG000001	2	protein_coding	protein-coding	flanking human accelerated region	regulatory region alteration	14
AGT	ENSG000001	1	protein_coding	protein-coding	human-specific derived allele	human-specific gene (undefined feature)	15
AH9	ENSG000001	6	protein_coding	protein-coding	human-specific accelerated evolution	gene sequence alteration	16,17
AHRR	ENSG000000	5	protein_coding	protein-coding	human-specific gene duplication	gene amplification	9
AL592528.1	ENSG000002	21	antisense	long non-coding	human-specific de novo originated protein-coding gene	de novo origin	8
AL772307.1	ENSG000001	9	processed_pseudogene	pseudogene	human-specific gene	human-specific gene (undefined feature)	6

HumanSpecific Genes | References | HumanSpecific GeneFamilies | +

Supplementary Table 1:
The screenshot above represents the first lines of the table. The full version is given as an independent Supplementary Table in xls format. This file contains all 845 genes with human-specific features retrieved in this study (**Sheet 1** - "HumanSpecific genes") and describes for each gene its: (A) Gene name (updated to the current Ensembl description, when necessary); (B) Ensembl ID; (C) Chromosome number; (D) Gene type (the specific type, as described by Ensembl); (E) General gene type (a general classification which may group multiple gene types - e.g. pseudogenes includes processed, unprocessed and transcribed pseudogenes); (F) Mechanism of origin (specifically as described by the author of the correspondent reference); (G) General mechanism of origin (a manually assigned general classification which may group multiple subclasses from column (F) - data from this column was used to generate the pie chart presented in Figure 1A in the main manuscript) and (H) At least one reference in which the gene is reported (the full list of references, numbered accordingly, is given as **Sheet 2** - "References"). The file also contains information on 19 large gene families (**Sheet 3** - "HumanSpecific GeneFamilies"), described as undergoing significant expansion or accelerated evolution across all (or many of) its members. These were not included in the main table, mainly to prevent their high gene numbers to introduce a functional bias in the dataset.

Supplementary Table 2 – Genomic Distribution of Genes with Human-Specific Features per Chromosome

Chromosome	% of protein-coding genes with human-specific features	% of protein-coding genes	P-value
X	11.41	4.15	4.50E-06
7	7.72	4.86	2.40E-03
5	2.85	4.36	0.012
15	2.52	2.99	0.013
10	6.38	3.6	0.016
8	2.68	3.31	0.041
2	7.55	6.38	0.1
16	4.70	4.27	0.11
Y	4.19	0.22	0.15
1	5.37	10.1	0.16
9	5.37	3.83	0.35
22	2.85	2.41	0.58
17	1.01	5.85	0.59
18	2.68	1.32	0.8
11	4.70	6.42	0.88
13	5.37	1.6	0.92
4	5.70	3.68	0.96
14	1.01	4.05	0.97
20	3.19	2.67	0.99
21	1.34	1.16	0.99
3	0.34	5.3	1
6	2.35	5.13	1
12	8.22	5.1	1
19	0.50	7.25	1

Supplementary Table 2:
This table presents the percentage of protein-coding genes in each chromosome (A), both for the set of genes with human-specific features (B) and the entire set of human proteins retrieved from the Ensembl database (C). A p-value is given (D), generated with a Fisher's exact test to represent the significance of the difference between (B) and (C) per chromosome. Chromosomes X and 7 are clearly enriched in genes with human-specific features and another four (in green) have significantly more of such genes than expected.

Supplementary Table 3 – Description of GeneTerm Linker Metagroups Comprising the Functional Networks on Supplementary Figure 1

MG	Silhouette	P-value	Genes	Terms / Exclusion criteria
1	-0.07	1.2e-52	145	Filtered out due to a silhouette size <0
2	0.15	6.6e-15	31	Blood vessel development (BP) GO<br>Camera-type eye development (BP) GO<br>Cerebellum development (BP) GO<br>DNA bending activity (MF) GO<br>Double-stranded DNA binding (MF) GO<br>Embryonic development (BP) GO<br>Embryonic heart tube development (BP) GO<br>Heart looping (BP) GO<br>Helix-loop-helix DNA-binding domain IPR<br>Negative regulation of transcription factor activity (BP) GO<br>Neural crest cell migration (BP) GO<br>Pattern specification process (BP) GO<br>Patterning of blood vessels (BP) GO<br>Regulation of transcription factor activity (BP) GO<br>Righting reflex (BP) GO<br>RNA polymerase II transcription factor activity, enhancer binding (MF) GO<br>Somitogenesis (BP) GO<br>Transcription factor binding (MF) * GO<br>Transcription factor complex (CC) GO<br>Transcription factor, fork head IPR
3`	0.00	2.6e-12	25	African trypanosomiasis KEGG<br>Cell adhesion molecules (CAMs) * KEGG<br>Cytokine-mediated signaling pathway (BP) GO<br>External side of plasma membrane (CC) * GO<br>Interferon-gamma-mediated signaling pathway (BP) GO<br>Leukocyte migration (BP) GO<br>Leukocyte transendothelial migration * KEGG<br>Malaria KEGG<br>Positive regulation of vasoconstriction (BP) GO<br>Regulation of immune response (BP) * GO<br>Response to ethanol (BP) GO<br>Staphylococcus aureus infection * KEGG<br>Transmembrane receptor activity (MF) GO
4`	0.13	3.1e-12	29	Alzheimer's disease KEGG<br>Artery morphogenesis (BP) GO<br>Cellular calcium ion homeostasis (BP) GO<br>Cholesterol homeostasis (BP) GO<br>Cholesterol metabolic process (BP) GO<br>Early endosome (CC) * GO<br>Intracellular transport (BP) * GO<br>Isomerase activity (MF) GO<br>Lipid homeostasis (BP) GO<br>Microtubule (CC) * GO<br>Peptidyl-prolyl cis-trans isomerase activity (MF) GO<br>Positive regulation of cholesterol efflux (BP) GO<br>Protein folding (BP) GO
5	0.20	1.7e-11	30	Basal cell carcinoma KEGG<br>Chromatin binding (MF) GO<br>DNA regulatory region binding (MF) GO<br>Drug binding (MF) GO<br>Enzyme binding (MF) GO<br>Nuclear hormone receptor, ligand-binding, core IPR<br>Positive regulation of NF-kappaB transcription factor activity (BP) GO<br>PPAR signaling pathway KEGG<br>Protein dimerization activity (MF) GO<br>Response to cold (BP) GO<br>Steroid hormone receptor IPR<br>Transcription factor binding (MF) * GO<br>Transcription, DNA-dependent (BP) GO<br>Zinc finger, nuclear hormone receptor-type IPR
6	-0.05	8.4e-11	31	Filtered out due to a silhouette size <0
7`	0.62	2.3e-10	16	Cadherin IPR<br>Calcium-dependent cell-cell adhesion (BP) GO<br>Homophilic cell adhesion (BP) GO<br>Synapse assembly (BP) GO
8`	0.12	3.6e-10	19	Calcium ion transport (BP) GO<br>Calcium signaling pathway * KEGG<br>Extracellular ligand-gated ion channel activity (MF) GO<br>Glutamatergic synapse KEGG<br>Glycine binding (MF) GO

				Ion channel activity (MF) * GO<br>Memory (BP) GO<br>Neuroactive ligand-receptor interaction * KEGG<br>Neurotransmitter-gated ion-channel IPR<br>Postsynaptic density (CC) GO<br>Postsynaptic membrane (CC) * GO<br>Synapse (CC) GO<br>Transporter activity (MF) * GO
9	0.01	7.6e-10	27	Activation of phospholipase C activity by G-protein coupled receptor protein signaling pathway coupled to IP3 second messenger (BP) GO<br>External side of plasma membrane (CC) * GO<br>GPCR, family 2-like IPR<br>GPCR, family 2, extracellular hormone receptor domain IPR<br>GPCR, family 2, secretin-like IPR<br>Ion transmembrane transport (BP) * GO<br>Muscle contraction (BP) GO<br>Neuroactive ligand-receptor interaction * KEGG<br>Neuropeptide signaling pathway (BP) GO<br>Postsynaptic membrane (CC) * GO<br>Protein localization (BP) GO<br>Trans-Golgi network (CC) GO
10	0.02	2.4e-09	34	Actin cytoskeleton (CC) GO<br>Apical plasma membrane (CC) GO<br>Axon terminus (CC) GO<br>Centriole (CC) GO<br>Centrosome (CC) * GO<br>Chromosome (CC) GO<br>Cilium (CC) GO<br>Condensed chromosome kinetochore (CC) GO<br>Hindbrain development (BP) GO<br>Microtubule cytoskeleton (CC) GO<br>Midbody (CC) GO<br>Mitosis (BP) * GO<br>Structural molecule activity (MF) GO
11`	0.22	9.6e-09	16	Axon guidance KEGG<br>Cell structure disassembly during apoptosis (BP) GO<br>Focal adhesion * KEGG<br>MAPK signaling pathway KEGG<br>Regulation of actin cytoskeleton KEGG<br>TGF-beta signaling pathway KEGG
12	0.16	2.4e-08	20	Antigen processing and presentation of peptide antigen via MHC class I (BP) GO<br>B302/SPRY domain IPR<br>Butyrophylin-like IPR<br>Cellular membrane organization (BP) GO<br>Cellular protein metabolic process (BP) GO<br>Early endosome (CC) * GO<br>Early endosome membrane (CC) GO<br>Endosome transport (BP) GO<br>ER to Golgi vesicle-mediated transport (BP) GO<br>Intracellular membrane-bounded organelle (CC) GO<br>Post-translational protein modification (BP) GO<br>Protein amino acid N-linked glycosylation via asparagine (BP) GO<br>SPla/RYanodine receptor subgroup IPR<br>SPRY-associated IPR<br>Zinc finger, RING-type IPR
13`	0.29	2.8e-08	18	AGC-kinase, C-terminal IPR<br>C2 calcium-dependent membrane targeting IPR<br>C2 membrane targeting protein IPR<br>C2 region IPR<br>Calcium signaling pathway * KEGG<br>Chemokine signaling pathway KEGG<br>Focal adhesion * KEGG<br>Gastric acid secretion KEGG<br>Leukocyte transendothelial migration * KEGG<br>Protein kinase C-like, phorbol ester/diacylglycerol binding IPR<br>Tight junction * KEGG<br>Vascular smooth muscle contraction KEGG
14	0.06	1.4e-07	15	External side of plasma membrane (CC) * GO<br>Growth factor activity (MF) GO<br>Hematopoietic cell lineage KEGG<br>Melanogenesis KEGG<br>Ovarian follicle development (BP) GO<br>Positive regulation of peptidyl-tyrosine phosphorylation (BP)

				GO
15`	0.33	1.6e-06	17	Fc gamma R-mediated phagocytosis KEGG<br>IgG binding (MF) GO<br>Leishmaniasis KEGG<br>Natural killer cell mediated cytotoxicity * KEGG<br>Osteoclast differentiation KEGG<br>Phagosome * KEGG<br>Staphylococcus aureus infection * KEGG<br>Systemic lupus erythematosus * KEGG<br>Tuberculosis KEGG
16`	0.21	1.1e-05	11	Natural killer cell mediated cytotoxicity * KEGG<br>Phagosome * KEGG<br>Regulation of immune response (BP) * GO<br>Staphylococcus aureus infection * KEGG<br>Systemic lupus erythematosus * KEGG
17`	0.29	1.2e-05	14	Cell adhesion molecules (CAMs) * KEGG<br>Leukocyte transendothelial migration * KEGG<br>Spermatid development (BP) GO<br>Tight junction * KEGG<br>Tight junction (CC) GO
18	0.73	2.4e-05	11	GRIP IPR<br>Intracellular transport (BP) * GO<br>Protein targeting to Golgi (BP) GO<br>Ran binding protein 1 IPR<br>Tetratricopeptide repeat IPR<br>Tetratricopeptide repeat-containing IPR
19`	0.16	5e-05	13	Dendrite (CC) GO<br>Neuroactive ligand-receptor interaction * KEGG<br>Neuronal cell body (CC) GO
20	0.33	0.00018	17	ABC transporter-like IPR<br>ABC transporter, integral membrane type 1 IPR<br>ABC transporters KEGG<br>ATPase activity (MF) GO<br>ATPase activity, coupled to transmembrane movement of substances (MF) GO<br>ATPase, AAA+ type, core IPR<br>Sodium ion transport (BP) GO<br>Transporter activity (MF) * GO
21	0.69	0.00024	11	GTPase activator activity (MF) GO<br>Rho GTPase-activating protein domain IPR
22	0.41	0.00037	10	Proteasome component (PCI) domain IPR<br>RNA transport KEGG<br>Translation initiation factor activity (MF) GO
23`	0.24	0.00043	12	Ion channel activity (MF) * GO<br>Ion transmembrane transport (BP) * GO<br>Transporter activity (MF) * GO
24`	0.30	0.00054	13	Chemokine activity (MF) GO<br>Chemokine interleukin-8-like domain IPR<br>Chemotaxis (BP) GO<br>Cytokine-cytokine receptor interaction KEGG<br>Inflammatory response (BP) GO<br>Jak-STAT signaling pathway KEGG<br>Neuroactive ligand-receptor interaction * KEGG
25	0.33	0.00059	14	Cell division (BP) GO<br>Centrosome (CC) * GO<br>G2/M transition of mitotic cell cycle (BP) GO<br>Microtubule (CC) * GO<br>Microtubule binding (MF) GO<br>Mitosis (BP) * GO<br>Mitotic cell cycle (BP) GO<br>Spindle pole (CC) GO
* Terms marked with an asterisk are in several Metagroups.<br>` Metagroups marked with an accent are depicted in Figure 1B in the main manuscript				

Supplementary Table 3:
This table describes the metagroups generated by GeneTerm Linker using FGNet. The metagroup number corresponds to numbers in Supplementary Figures 1A and 1B (and information can be transferred to Figure 1B in the manuscript). For each metagroup this file presents its silhouette size (a clustering coefficient), significance (p-value), number of constituent genes and constituent functional terms (or, for metagroups 1 and 6, which do not meet inclusion cutoffs, their exclusion criteria). The last column on the right describes functional terms in each metagroup and their annotation space, which can be a gene ontology assignment (GO for biological process, molecular function or cellular component), a KEGG pathway or a function inferred from the description of InterPro motifs or domains (IPR).

Supplementary Table 4 – Expression levels of transcripts produced by human-specific genes in subpopulations of glial cells

(See Below)

Supplementary Table 4:
This table presents gene expression levels (in FPKM) for transcripts related to 61 human-specific genes which were characterized as highly expressed in at least one subpopulation of glial cell (sequencing data retrieved from SRA). Highly expressed transcripts were defined as the top ~10% of the expressed transcripts (i.e. the 2,000 transcripts) with highest average FPKM values for each set of samples under investigation. On average ~1580 genes were characterized as highly expressed in each cell type and compared with the set of 856 genes with human-specific features. We retrieved 23 highly expressed human-specific genes from the radial glial cell samples, 17 from the outer radial glial cell samples, 26 from the progenitor cells samples and 24 from the neuron cell samples, resulting in a set of 61 non-redundant genes and 91 transcripts.

Gene ID	Gene Name	Gene Type	Transcript ID	Expression in FPKM			
				RG	oRG	IPC	Neuron
ENSG00000128185	DGCR6L	protein_coding	ENST00000248879	35.0375	35.1325	65.785	2.6
ENSG00000131051	RBM39	protein_coding	ENST00000253363	97.235	27.1025	150.645	58.045
ENSG00000154016	GRAP	protein_coding	ENST00000284154	96.785	0.0775	0	0
ENSG00000163956	LRPAP1	protein_coding	ENST00000296325	203.52	361.44	64.3225	0.196667
ENSG00000166803	KIAA0101	protein_coding	ENST00000300035	98.1675	107.323	0.0975	0.0666667
ENSG00000167977	KCTD5	protein_coding	ENST00000301738	19.77	0.295	88.51	33.735
ENSG00000143742	SRP9	protein_coding	ENST00000304786	172.805	105.73	184.972	327.2
ENSG00000172239	PAIP1	protein_coding	ENST00000306846	0.035	0	0.0875	91.66
ENSG00000125462	C1orf61	protein_coding	ENST00000310027	34.495	267.295	0	14.1767
ENSG00000176476	CCDC101	protein_coding	ENST00000317058	139.74	74.38	0.2475	0
ENSG00000144218	AFF3	protein_coding	ENST00000317233	0.0325	0	6.7575	71.8017
ENSG00000076382	SPAG5	protein_coding	ENST00000321765	0.79	0	142.222	0.0516667
ENSG00000186868	MAPT	protein_coding	ENST00000334239	0	0	0	66.3583
ENSG00000176809	LRRC37A3	protein_coding	ENST00000339474	100.13	0	22.0275	0.0816667
ENSG00000150991	UBC	protein_coding	ENST00000339647	89.79	316.275	207.647	287.858
ENSG00000146872	TLK2	protein_coding	ENST00000343388	0.1325	47.11	31.04	70.755
ENSG00000142330	CAPN10	protein_coding	ENST00000352879	67.1225	14.75	76.2625	38.5017
ENSG00000196937	FAM3C	protein_coding	ENST00000359943	74.605	0	27.4625	54.2933
ENSG00000198826	ARHGAP11A	protein_coding	ENST00000361627	10.14	0.5525	179.1	0.0633333
ENSG00000117650	NEK2	protein_coding	ENST00000366999	0.0975	0	394.72	0.451667
ENSG00000066279	ASPM	protein_coding	ENST00000367408	0.03	0.035	199.335	0.153333
ENSG00000066279	ASPM	protein_coding	ENST00000367409	0	0	67.8525	0.105
ENSG00000125462	C1orf61	protein_coding	ENST00000368243	4.225	76.55	0	0
ENSG00000265241	RBM8A	protein_coding	ENST00000369307	0	19.01	135.275	35.845
ENSG00000188610	FAM72B	protein_coding	ENST00000369390	0	0	77.6275	0
ENSG00000148308	GTF3C5	protein_coding	ENST00000372099	86.97	0	0	6.485
ENSG00000175170	FAM182B	protein_coding	ENST00000376404	73.255	0	0	0
ENSG00000132406	TMEM128	protein_coding	ENST00000382753	0.1725	119.58	8.795	108.818
ENSG00000141562	NARF	protein_coding	ENST00000390006	0	0	104.927	24.7217
ENSG00000241973	PI4KA	protein_coding	ENST00000399213	41.2725	10.115	8.32	56.325
ENSG00000125462	C1orf61	protein_coding	ENST00000400991	71.11	482.913	35.7175	112.928
ENSG00000077380	DYNC1I2	protein_coding	ENST00000409197	0.19	98.2775	177.692	0.808333
ENSG00000152076	CCDC74B	protein_coding	ENST00000409943	96.2325	0	0	0
ENSG00000077380	DYNC1I2	protein_coding	ENST00000410079	0.18	24.2225	73.7925	23.39
ENSG00000197620	CXorf40A	protein_coding	ENST00000423421	0	84.1375	0	79.2817
ENSG00000197620	CXorf40A	protein_coding	ENST00000423540	0	0	0	67.1217
ENSG00000219481	NBPF1	protein_coding	ENST00000430580	29.7625	30.6825	71.01	50.1583
ENSG00000122545	SEPT7	protein_coding	ENST00000432293	67.1525	140.39	135.148	95.235
ENSG00000136861	CDK5RAP2	protein_coding	ENST00000433194	0.27	45.0875	11.215	148.2
ENSG00000122545	SEPT7	protein_coding	ENST00000435235	29.61	232.685	292.915	72.6783
ENSG00000130305	NSUN5	protein_coding	ENST00000438747	30.235	2.99	27.45	72.735
ENSG00000166086	JAM3	protein_coding	ENST00000441717	101.55	26.575	0.7575	1.1
ENSG00000131051	RBM39	protein_coding	ENST00000444878	100.245	0	16.005	0
ENSG00000186868	MAPT	protein_coding	ENST00000446361	36.7275	0	0	58.3133
ENSG00000235597	LINC01102	lincRNA	ENST00000447380	31.0375	0	35.275	124.353
ENSG00000219481	NBPF1	protein_coding	ENST00000449853	0.035	0	11.3125	83.3
ENSG00000143702	CEP170	protein_coding	ENST00000451408	0	0	18.0875	86.1533

ENSG00000082684	SEMA5B	protein_coding	ENST00000451541	63.57	98.5325	0.07	0.151667
ENSG00000130305	NSUN5	protein_coding	ENST00000455763	0	0	1.62	69.125
ENSG00000131051	RBM39	protein_coding	ENST00000461283	11.445	76.815	143.235	55.2917
ENSG00000197021	CXorf40B	protein_coding	ENST00000462691	73.3975	0	0	36.1167
ENSG00000257267	ZNF271P	unitary_pseudogene	ENST00000465539	0.1	29.9775	4.18	70.0783
ENSG00000143702	CEP170	protein_coding	ENST00000468254	0.0325	68.28	234.855	266.102
ENSG00000134899	ERCC5	protein_coding	ENST00000472247	0	0	77.26	15.0317
ENSG00000077380	DYNC1I2	protein_coding	ENST00000479806	63.9	34.56	69.395	144.263
ENSG00000125462	C1orf61	protein_coding	ENST00000484428	120.882	0	24.2025	0
ENSG00000075292	ZNF638	protein_coding	ENST00000487638	58.5775	0	29.5575	60.615
ENSG00000131051	RBM39	protein_coding	ENST00000496183	7.6625	45.0975	2.32	56.66
ENSG00000125462	C1orf61	protein_coding	ENST00000497824	332.195	271.06	52.5625	140.453
ENSG00000143126	CELSR2	protein_coding	ENST00000498157	70.0225	1.71	0.6875	38.5617
ENSG00000125462	C1orf61	protein_coding	ENST00000498346	152.225	50.37	19.505	57.55
ENSG00000249915	PDCD6	protein_coding	ENST00000507528	0	0	106.448	0
ENSG00000073578	SDHA	protein_coding	ENST00000509564	3.3525	0.495	64.405	7.16667
ENSG00000145725	PPIP5K2	protein_coding	ENST00000509597	5.25	45.845	6.66	124.932
ENSG00000135541	AHI1	protein_coding	ENST00000524469	87.865	47.3275	0	0.41
ENSG00000137812	CASC5	protein_coding	ENST00000526913	0	16.2575	92.8	0.0416667
ENSG00000137812	CASC5	protein_coding	ENST00000531626	0	16.35	238.06	1.03
ENSG00000148737	TCF7L2	protein_coding	ENST00000534894	0	78.7	0	0
ENSG00000172915	NBEA	protein_coding	ENST00000537702	0	0	0	74.32
ENSG00000139372	TDG	protein_coding	ENST00000544060	94.4075	27.16	0	18.77
ENSG00000150991	UBC	protein_coding	ENST00000544481	19.88	113.353	41.98	28.5333
ENSG00000150991	UBC	protein_coding	ENST00000546120	45.935	82.28	62.375	122.56
ENSG00000123064	DDX54	protein_coding	ENST00000549271	71.25	0	43.1075	0.15
ENSG00000123064	DDX54	protein_coding	ENST00000550016	105.697	0	0	0
ENSG00000165525	NEMF	protein_coding	ENST00000555970	0	0	67.0975	19.1383
ENSG00000185650	ZFP36L1	protein_coding	ENST00000555997	32.1725	130.873	0	0
ENSG00000166803	KIAA0101	protein_coding	ENST00000558043	85.665	159.188	0	0.13
ENSG00000166803	KIAA0101	protein_coding	ENST00000559519	99.265	188.282	0	0
ENSG00000198826	ARHGAP11A	protein_coding	ENST00000562481	0	0.3375	246.488	0
ENSG00000205336	GPR56	protein_coding	ENST00000564907	131.873	74.7225	11.4225	0.0616667
ENSG00000198826	ARHGAP11A	protein_coding	ENST00000565905	0	0	256.26	0
ENSG00000140365	COMMD4	protein_coding	ENST00000566843	80.5675	0.345	0.67	0.635
ENSG00000140365	COMMD4	protein_coding	ENST00000567195	0	0	108.877	0
ENSG00000120071	KANSL1	protein_coding	ENST00000576870	4.3125	5.84	17.5525	79.6517
ENSG00000076382	SPAG5	protein_coding	ENST00000582076	21.925	0	162.292	0.19
ENSG00000187951	ARHGAP11B	protein_coding	ENST00000602616	36.78	0	99.8325	0
ENSG00000272398	CD24	protein_coding	ENST00000606017	27.57	146.417	1584.35	2219.95
ENSG00000066279	ASPM	protein_coding	ENST00000612785	0.02	0.1275	115.62	0.261667
ENSG00000128408	RIBC2	protein_coding	ENST00000614167	155.197	0.1	0	0.0933333
ENSG00000249915	PDCD6	protein_coding	ENST00000614778	161.93	0	8.055	46.865
ENSG00000125462	C1orf61	protein_coding	ENST00000615748	217.755	139.843	30.4325	0

Supplementary Table 5 – Primers Used for RT-qPCR

Gene Name	Forward Primer (5'- 3')	Reverse Primer (5' - 3')	Accession #
TENM1	GAACCCACCTACACACCCTG	CCATTGCTGCTGGTAATCGC	NM_001163279.1
CAPN1	GGCCAGGATTATGAGCAGCT	GGTCCTTGTAACCCAGGCTC	NR_040008.1
FAM21A	GCAGGAGAAGACACGAGAGC	TTGCTCAAAGGCACTGTCCA	NM_001330102.1
SLC7A6	GCGCTCATTGCCATCATTGT	GTTTCCCATGTCCCAGGAGG	NM_001076785.2
SEPT7	GGACTCTGAAGCTGAGCTCC	CATCCTCGAACTGACGACGT	NM_001788.5
RBM39	ATCTCTTCCCGAACACGAGC	GGAGCCTCAAGCATTGCTTC	NR_136587.1
KIAA0319L	AGAGAAGGGCAAACTCCTGC	ACCAGGTGGCTACAGGATCT	NM_024874.4
AFF3	CTCCGGGAATGATCAGAGGC	GATGTCACTGGTGGTCCTGG	NM_002285.2
PPIP5K2	ATGAAGAGAGCCCCCTGAGT	GTTCCCCTGATCTGCGTCTT	NM_015216.4
TLK2	TGGAGCTGAAATGGGAGACG	CGACCACATGTTCAGGCTCT	NM_001330418.1
NLGN4X	GCAATGGGCTGCCAAGAAAA	GGCTTTCCAGGGAGCAGTAG	NM_001282146.1
ZNF286A	TCGATCGTCCGATTCTCCCA	GCCCGGAACTACAATTCCCA	NM_001288645.1

Supplementary Table 5:
Primers designed for each of the 12 genes described in the Figure 4 of the main manuscript. Gene names and accession numbers are also provided.
